# Combined Electrospinning–Electrospraying for High-Performance Bipolar Membranes with Incorporated MCM-41 as Water Dissociation Catalysts

**DOI:** 10.1021/acsami.3c06826

**Published:** 2023-09-20

**Authors:** Emad Al-Dhubhani, Michele Tedesco, Wiebe M. de Vos, Michel Saakes

**Affiliations:** † Wetsus, European Centre of Excellence for Sustainable Water Technology, Oostergoweg 9, 8911 MA Leeuwarden, The Netherlands; ‡ Membrane Science and Technology, University of Twente, P.O. Box 217, 7500 AE Enschede, The Netherlands

**Keywords:** electrospinning–electrospraying, bipolar membrane, water dissociation, catalyst, MCM-41

## Abstract

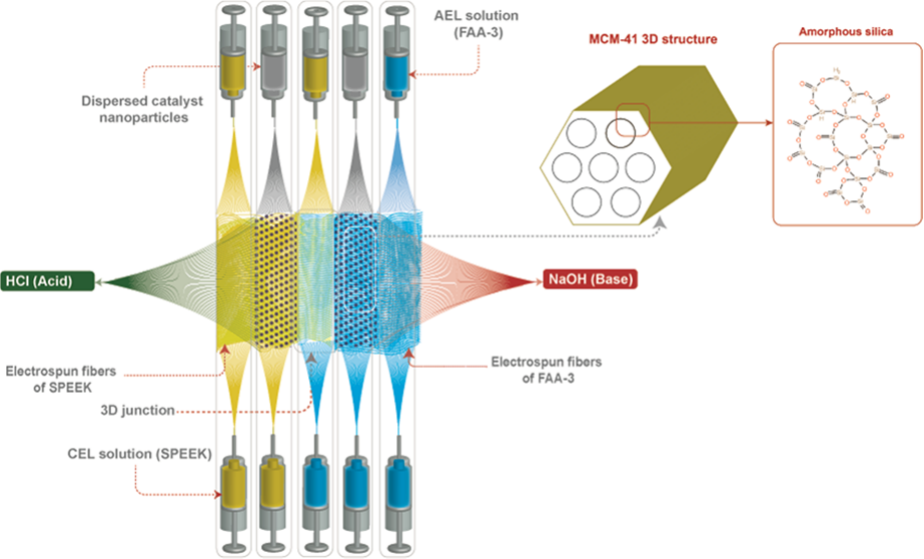

Electrospinning has been demonstrated as a very promising method to create bipolar membranes (BPMs), especially as it allows three-dimensional (3D) junctions of entangled anion exchange and cation exchange nanofibers. These newly developed BPMs are relevant to demanding applications, including acid and base production, fuel cells, flow batteries, ammonia removal, concentration of carbon dioxide, and hydrogen generation. However, these applications require the introduction of catalysts into the BPM to allow accelerated water dissociation, and this remains a challenge. Here, we demonstrate a versatile strategy to produce very efficient BPMs through a combined electrospinning–electrospraying approach. Moreover, this work applies the newly investigated water dissociation catalyst of nanostructured silica MCM-41. Several BPMs were produced by electrospraying MCM-41 nanoparticles into the layers directly adjacent to the main BPM 3D junction. BPMs with various loadings of MCM-41 nanoparticles and BPMs with different catalyst positions relative to the junction were investigated. The membranes were carefully characterized for their structure and performance. Interestingly, the water dissociation performance of BPMs showed a clear optimal MCM-41 loading where the performance outpaced that of a commercial BPM, recording a transmembrane voltage of approximately 1.11 V at 1000 A/m^2^. Such an excellent performance is very relevant to fuel cell and flow battery applications, but our results also shed light on the exact function of the catalyst in this mode of operation. Overall, we demonstrate clearly that introducing a novel BPM architecture through a novel hybrid electrospinning–electrospraying method allows the uptake of promising new catalysts (i.e., MCM-41) and the production of very relevant BPMs.

## 1 Introduction

Bipolar membranes (BPMs) are a unique type of ion exchange membrane that combines cation exchange and anion exchange layers. A key property of BPMs is their ability to dissociate water molecules into hydroxide ions (OH^–^) and protons (H^+^) when subjected to an electrical current in an electrochemical system. While this makes them relevant to produce acids and bases, they have also been successfully applied for zero-gap bipolar membrane water electrolysis. Such BPM-based systems for hydrogen gas formation combine advantages such as a high rate of hydrogen evolution and low cost-efficient oxygen evolution using non-noble metals-based electrocatalysts as anodes in an alkaline environment.^1−5^ Moreover, BPMs can be a core component for flow batteries, where they generate acid and base from water during charging and recombine them into water during discharging.^6−13^

BPMs possess a versatile nature, allowing their use in different processes and (environmental) applications. For instance, recent studies focused on integrating BPMs in electrolysis-based processes for CO_2_ reduction.^14−17^ Moreover, BPM applications extend into fields such as the recovery of lithium and boron,^18,19^ desalination of saline water,^20^ recovery of ammonia,^21−23^ and bipolar membrane-based fuel cells.^24^

Water dissociating catalysts are a critical component of any BPM. They are integrated into the BPM to improve the efficiency and rate of water dissociation. Many different types of materials were investigated for their capabilities of assisting water dissociation in a BPM. Sebatian et al. investigated around 40 different metal/metal oxide nanoparticles with the intention of combining catalyst efficient near acidic conditions in the cation exchange layer (CEL) with catalysts that operate efficiently under basic conditions in the anion exchange layer (AEL). Here, BPMs that combined precious metal catalysts like Ir/TiO_2_ (AEL) and RuO_2_ or IrO_2_ (CEL) led to a superior performance.^25^

Said et al. fabricated BPMs with a thin polyelectrolyte layer at the interface. They used the approach of layer-by-layer assembly to coat the interface with poly(3,4-ethylenedioxythiophene):poly(styrenesulfonate) (PEDOT:PSS) and poly(ethylenimine) (PEI), leading to an enhancement in the water splitting performance and a positive impact on selectivity. Additional studies reported the utilization of other polymeric materials to improve the BPM water dissociation capabilities, such as Boltorn,^26^ poly(ethylene glycol) (PEG),^27^ and poly(4-vinylpyrrolidone) (P4VP).^7^

Still, even more types of catalytic materials have been used in the fabrication of BPMs. McDonald et al. showed that reduced graphene oxide (r-GO) lowered the ionic resistance of water dissociation in a BPM.^28^ Additionally, MIL-101,^29^ Nano-MoS_2_,^30^ Fe complex,^31^ and Palygorskite^32^ were effective in lowering the water dissociation potential across the BPM. The most common techniques of introducing catalysts into the BPM matrix were air-spraying, solution casting, and dip/spin-coating. Table 1 shows some characteristics of recently reported BPMs in the literature.

**Table 1:** Characteristics and Performance of Recently Reported BPMs

BPM	method	water dissociation catalyst used	loading	performance	reference
SCBM	shielding and in situ formation strategy	goethite Fe^3+^O(OH)		1.1 V@1000 A/m^2^	(33)
PEI-based BPM	solution casting	Fe(III)@PEI-based BPM		1.88 V@3200 A/m^2^	(34)
SBM-NC2.0	casting	montmorillonite nanoclay	0.25–2 mg/cm^2^	1.1 V@500 A/m^2^	(35)
NIA-2.28	micropatterning	Al(OH)_3_	0.02–0.5 mg/cm^2^	3.25 V@500 A/m^2^	(36)
BPM	atomic layer deposition (ALD)	IrO_2_ in acid and NiO in base		1.9 V@1000 A/m^2^	(25)
BPM_VO-ns	solution casting	V_2_O_5_-nanosheets blended with poly(vinyl alcohol) (PVA)		3.6 V@1000 A/m^2^	(37)

**Figure 1:**
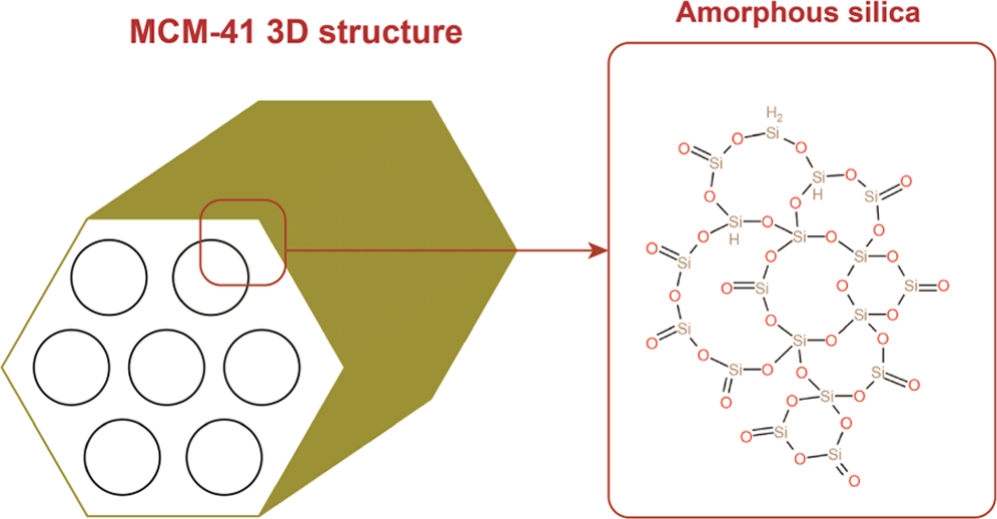
Structure of nanosized silica MCM-41.

In this study, we propose a new catalyst for BPMs, MCM-41 (Figure 1). This catalyst possesses a mesoporous structure of a zeolitic-like framework of amorphous silica with a very large specific surface area. MCM-41 was reported to have a specific surface area in the range of 1000–1500 m^2^/g and an average pore diameter between 2.5 and 3.6 nm.^38,39^ MCM-41 has been widely used in different applications, both in its pure form or transition metal modified form, including catalytic cracking,^40,41^ a photocatalysis for hydrogen evolution,^42,43^ organic pollutant degradation,^44^ CO_2_ sorption,^45^ and the fabrication of membranes for polymer electrolyte fuel cells.^46^

In this work, MCM-41 nanoparticles were introduced as catalyst materials for water dissociation in electrospun BPM. This work involves a novel BPM architecture approach that allows incorporation of the catalyst into layers directly adjacent to the three-dimensional (3D) junction of entangled cation exchange and anion exchange nanofibers. To achieve this, we use a dual fabrication approach, electrospinning the polymeric materials and electrospraying the catalytic nanoparticles. This method of hybrid fabrication will be shown to be advantageous for several reasons, such as overcoming the incompatibility issues of blending inorganic catalyst nanoparticles and polymeric materials during fabrication, flexibility of introducing higher catalyst loading without compromising the integrity of the membrane structure, and reducing waste materials during the process of fabrication.

In this research work, six bipolar membranes were fabricated using our dual electrospinning–electrospraying approach. Four of these had different catalyst (MCM-41) loadings, while two BPMs were fabricated by introducing the catalyst at only one side of the 3D junction (anion exchange side and cation exchange side). The fabricated BPMs were then studied by various morphological and electrochemical characterizations, including their potential for water dissociation and association.

## 2 Materials and Methods

### 2.1 Materials and Reagents

The materials used for this work, including the anion exchange polymer Fumasep (FAA-3) and the cation exchange polymer of sulfonated poly(ether ether ketone) (SPEEK), were purchased from Fumatech BWT GmbH (Germany). The polymeric catalysts poly(4-vinylpyrrolidone) (P4VP) with a molecular weight of 60,000 g/mol were purchased from Sigma-Aldrich as a dry powder. MCM-41 was utilized as a water dissociation catalyst in the form of nanoparticles with a hexagonal mesoporous structure as acquired from Sigma-Aldrich with the size of 2–4 nm. Polymers were dissolved in dimethylacetamide (DMAc) as the solvent (VWR Chemicals). All of the materials were used as received.

### 2.2 Membrane Fabrication through Dual Electrospinning/Electrospraying

**Figure 2:**
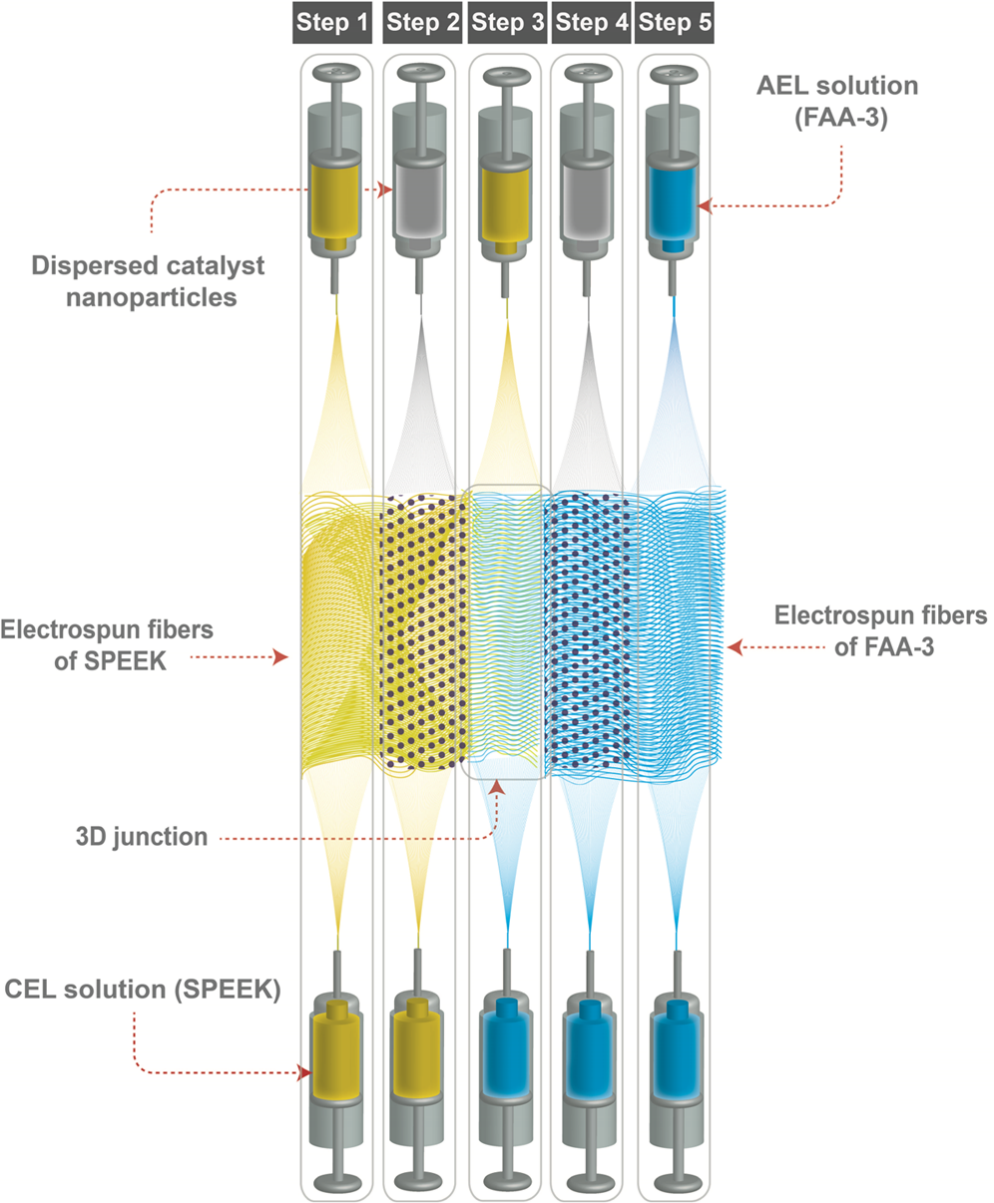
Illustration of the fabrication approach of combined electrospinning–electrospraying, the orientation of the electrospinning/electrospraying orifices appears to be in parallel to the plane only for the sake of demonstrating the sequence of fabrication and not the actual orientation.

The methodology of fabricating BPMs using an electrospinning/hot-pressing approach has been thoroughly reported in our previous study.^7^ The manufacturing of the BPM was performed in six main consecutive stages (Figure 2): first, the cation exchange polymer (SPEEK) was electrospun simultaneously from the two dispensers in the electrospinning setup of LE-50 from Bioinicia (Spain). Then, one dispenser role was exchanged to electrospray an MCM-41 catalyst dispersion of 2% in water/ethanol (90:10) ratio while maintaining the other dispenser to continue electrospinning of SPEEK. This step ensures dispersion and distribution of MCM-41 nanoparticles within the matrix of CEM (SPEEK) nanofibers. Following that, the catalyst dispensing was exchanged with FAA-3 electrospinning for the building up of the 3D junction of entangled SPEEK and FAA-3 fibers, just as the ordinary dual electrospinning reported in early work.^7^ Important to mention here is that the water splitting catalyst MCM-41 is not present in this 3D junction layer. Expanding the investigation into including the catalyst (MCM-41) in the 3D junction requires a setup that possesses three nozzles. Such a setup could simultaneously electrospin/electrospray from three different solutions, thus allowing 3D junction fabrication and inclusion of catalyst. In the following stage, the electrospinning continued with building up the AEL. At this stage, anion exchange polymer (FAA-3) was electrospun simultaneously with electrospraying of MCM-41 nanoparticles. However, the nanoparticles were dispersed in the AEL nanofibers, in the same way as the structure that was formed in step 2. The duration of electrospraying MCM-41 at a constant rate and a known solution concentration determines the nanoparticle loading. Following that, the anion exchange polymer (FAA-3) was electrospun simultaneously from the two dispensers. Finally, a hot-pressing procedure was performed to transform the porous electrospun mat into a dense BPM. The porous electrospun mat was placed between two PTFE sheets, which were in turn sandwiched between two stainless steel plates. They were subjected to an hour-long hot-pressing at 150 °C and 200 bar.

The described fabrication procedure is illustrated in Figure 2, while Table 2 summarizes the parameters of electrospinning and electrospraying. Table 3 lists the different fabricated BPMs in this work by the location of the catalyst and catalyst loading. Four BPMs were fabricated with different loadings and with catalysts on both sides of the 3D junction. Additionally, two BPMs were fabricated with the catalyst introduced to the cation exchange side only and to the anion exchange side only.

**Table 2:** Summary of All Electrospinning and Electrospraying Parameters

		electrospinning		electrospraying
		anion exchange materials	cation exchange materials	catalysts nanoparticles
	unit	FAA-3/P4VP	SPEEK	MCM-41
solvent		dimethylacetamide, DMAc		dispersion in water/ethanol (90:10)
concentration	wt %	26	20	2
temperature	°C	30	30	30
relative humidity	wt %	20	20	20
drum speed	rpm	200	200	200
syringe diameter	mm	20.05		
drum voltage	kV	–10		
tip voltage	kV	+6	+18	+10
flow rate	mL/h	0.5	0.7	1
distance tip to collector	mm	100	75	150

**Table 3:** List of the BPMs Fabricated in This Work by Configuration and MCM-41 Catalyst Loading

bipolar membrane name	duration of nanosprayed catalyst deposition (h)	location of catalyst	MCM-41 catalyst loading (mg/cm^2^)
BPM-0.5 h	0.5	both CEL and AEL (both sides of the 3D junction)	0.07
BPM-1 h	1		0.13
BPM-2 h	2		0.27
BPM-4 h	4		0.53
BPM-1 h-CES	1	CEL side only	0.07
BPM-1 h-AES	1	AEL side only	0.07

### 2.3 Scanning Electron Microscope (SEM) Analysis and Elemental Mapping (EDX)

Cross-sectional imaging was conducted using a scanning electron microscope (SEM) equipped with an energy-dispersive X-ray analysis (EDX) system (JEOL JSM-6480 LV). Cross-sectional imaging was used to examine the hot-pressed electrospun membranes to provide an estimation of the layer dimensions. Membranes were pretreated in 1 M NaCl solution prior to the drying stage, during which water was removed by placing the membranes in a vacuum oven at 50 °C overnight.

### 2.4 Electrochemical Characterization

**Figure 3:**
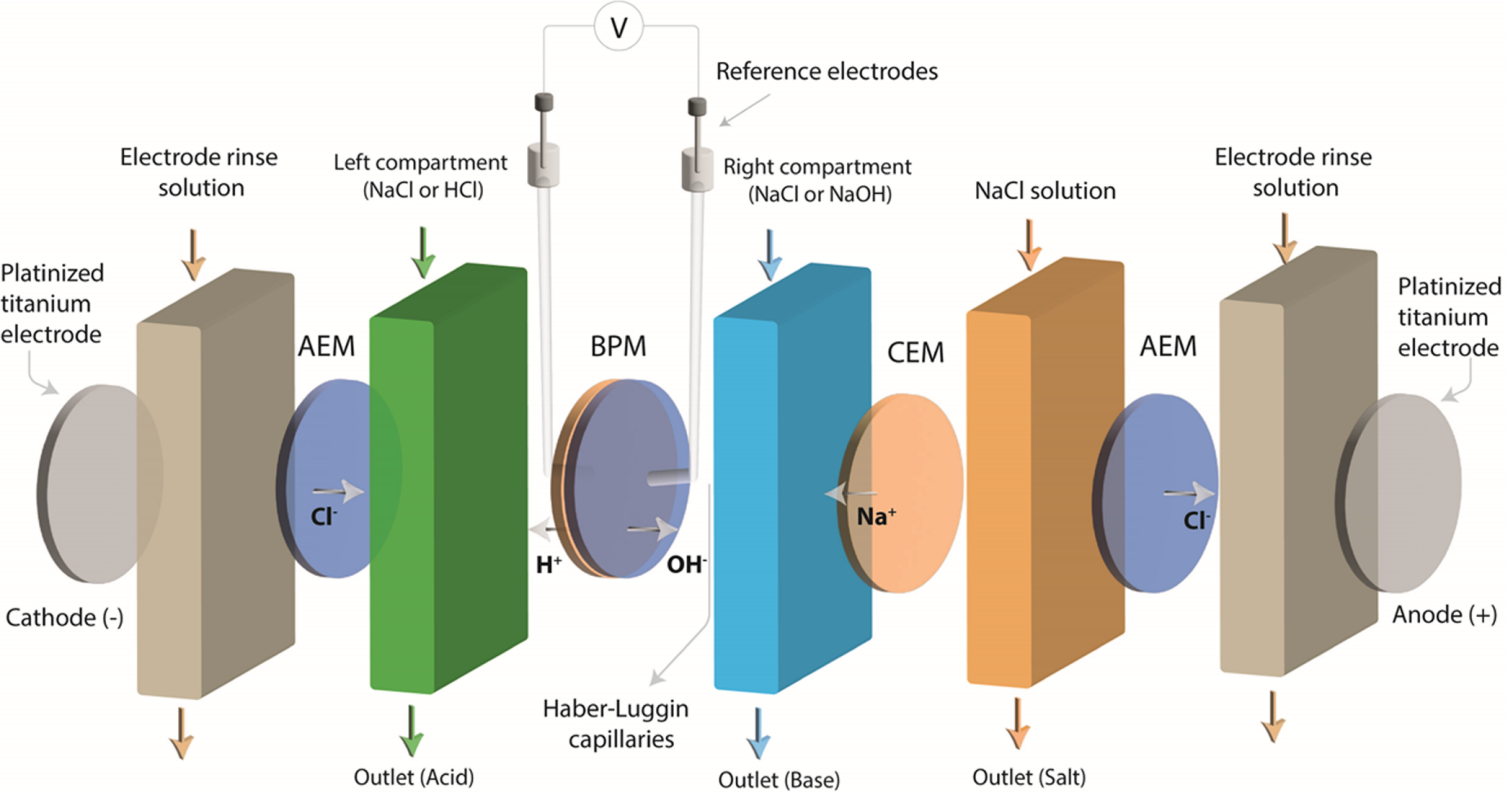
Five-compartment acid–base flow battery setup equipped with Luggin capillaries with Ag/AgCl reference electrodes for the *I*–*V* measurements of the BPMs using two platinized Ti electrodes.

The electrochemical characterization of the bipolar membranes was performed using a homemade five-compartment poly(methyl methacrylate) (PMMA) testing cell (see Figure 3). Each compartment was separated by a different ion exchange membrane with an active membrane area of 7 cm^2^ by placement of the bipolar membrane between two plastic insert plates with circular holes. Furthermore, the setup consisted of two platinized titanium electrodes (2.5 μm Pt) (Magneto Special Anodes, Schiedam, The Netherlands) placed in the electrode compartments. Two Haber–Luggin capillaries were positioned at both sides of the BPM and connected to two Ag/AgCl reference electrodes (3 M KCl; QM711X, QIS, The Netherlands) to measure the voltage drop across the bipolar membrane. The reference electrodes were connected to the sense and reference electrodes of a potentiostat (IviumStat.XRi, Ivium Technologies, The Netherlands) for registration of the voltage drop. The platinized titanium electrodes were connected to the working and counter electrodes of the same Ivium potentiostat for employing an electrical current. The electrode rinse solution of the anode and cathode consisted of 0.25 M iron(II) chloride and 0.25 M iron(III) chloride. All solutions were circulated at a rate of 400 mL/min through the cell compartments using Masterflex pumps.

### 2.5 Current Efficiency and Energy Consumption

Current efficiency and energy consumption of acid and base production were measured in 0.5 M NaCl solution by recording the pH change of the acid or base compartment. Based on our experience, the pH is more stable in the base compartment and less stable in the acid compartment due to the high mobility of H^+^ ions and the co-ion transport of H^+^ ions through the anion exchange membranes. Thus, the pH was measured for the recirculating electrolyte of the base compartment. The current efficiency and the energy consumption were calculated following the equations given below:
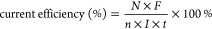
1where *N* is the molar equivalent of hydrochloric acid, *n* is the number of bipolar membranes (*n* = 1 for this system), *F* is the Faraday constant (96,485 C/mol), *I* is the current (A), and *t* (s) is the time of the experiment (water dissociation).
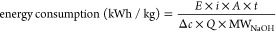
2where *E* is the voltage (in V) across the BPM, *i* is the current density (in A/m^2^) applied, *A* is the active area of BPM (in m^2^), *t* is the time (in s) of the process, Δ*c* the concentration (in mol/L) change of NaOH, *Q* is the volume of water (in m^3^) recirculated, and MW_NaOH_ is the molecular weight of NaOH (39.99 g/mol). Current efficiency and energy consumption enable comparison of the bipolar membranes in terms of the water dissociation (i.e., production of acid and base) at a given current density. In this work, the production of acid and base was tested under galvanostatic conditions at current densities of 100, 200, and 400 A/m^2^ to compare the fabricated BPMs.

## 3 Results and Discussion

### 3.1 Morphological Characterization of Fabricated BPMs

**Figure 4:**
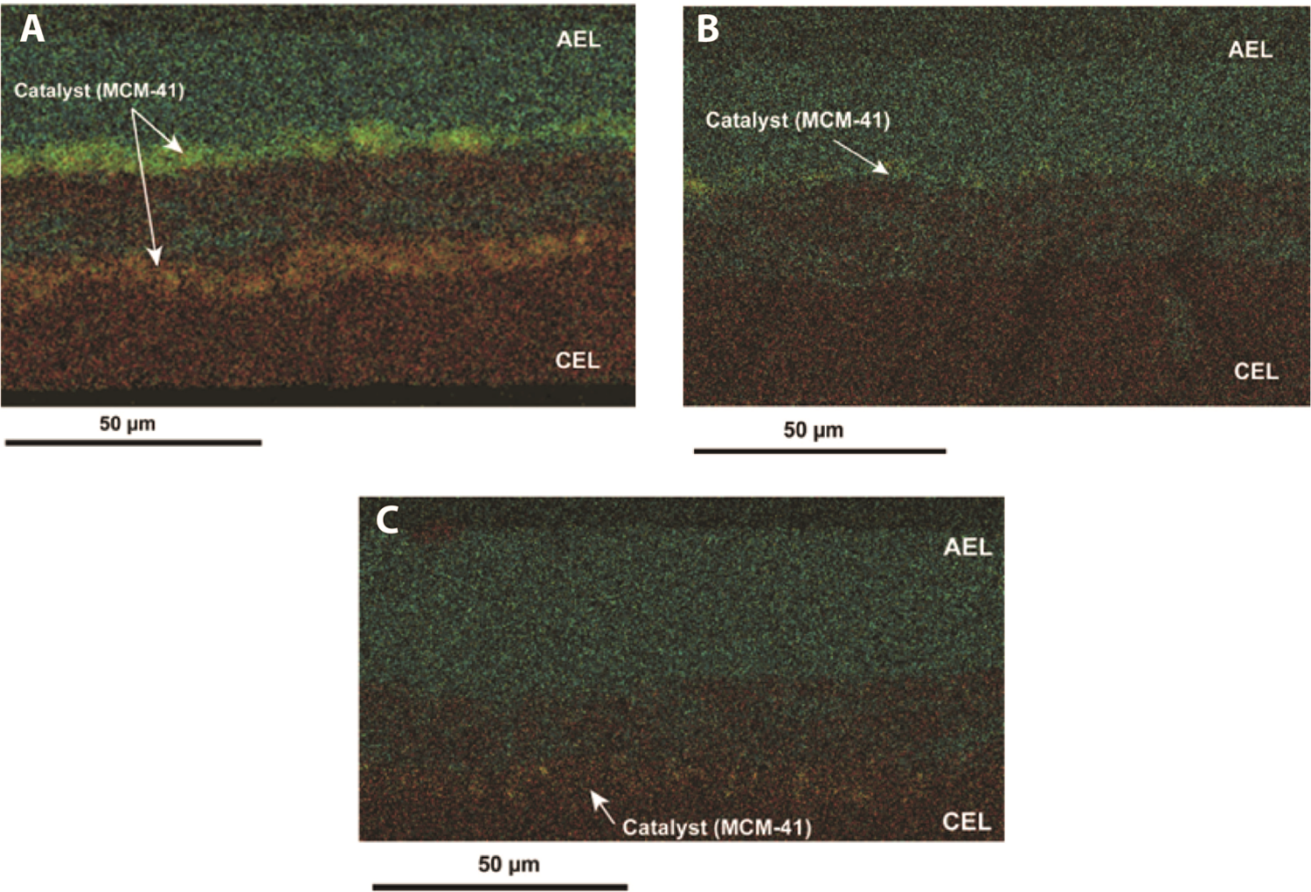
Cross-sectional SEM–EDX images of fabricated BPMs: (A) BPM-2 h, (B) BPM-1 h-AES, and (C) BPM-1 h-CES. Elemental mapping for sulfonate ions (red), bromide Br ions (green), and Silicon Si (yellow). Imaging of dense BPM films was performed after the hot-pressing step.

To check if the formed membranes obtained the desired structure, SEM–EDX was used, and the resulting cross-sectional SEM–EDX images are shown in Figure 4. Initially, we focus on Figure 4A, where the MCM-41 nanoparticles were electrosprayed for 2 h on both sides of the 3D junction. The composition of the images demonstrates an equivalent number of layers, as anticipated from the fabrication approach. Those layers are color-coded: green regions are associated with bromide ions (anion exchange polymer, FAA-3), yellow regions are linked to the excitation of silicon atoms stemming from the introduced nanostructured nanoparticles (MCM-41), while the red color-coded regions correspond to sulfonate ions, a chemical group in the cation exchange polymer (SPEEK). Figure 4A verifies the placement of MCM-41 in distinct layers adjacent to the central 3D junction. The 3D junction composition, giving signals of both cation and anion exchange polymers, aligns with the previously described work. Clearly, complex layered materials can be produced by our proposed combination of electrospinning and electrospraying, allowing the design of novel BPM architectures.

In Figure 4B,C, we show the SEM–EDX results for BPMs fabricated with MCM-41 only electrosprayed to one side of the BPM 3D junction. The BPM formed with MCM-41 electrosprayed at the anion exchange side is designated as (BPM-1 h-AES, 4B), while the designation of (BPM-1 h-CES, 4C) refers to BPM with the catalytic nanoparticles introduced to the cation exchange side. The figures show clear yellow regions on the expected side of the anion 3D junction. The estimated thicknesses of the layers are 30, 20, and 30 μm for AEL, 3D junction, and CEL, respectively. The fabrication process follows the guidance reported previously, which tunes the flow rate and duration of deposition to achieve these thicknesses. Additionally, deposition of catalyst (MCM-41) nanoparticles does not have a major impact on the thickness of the layer.

### 3.2 Water Dissociation Electrochemical Characterization

**Figure 5:**
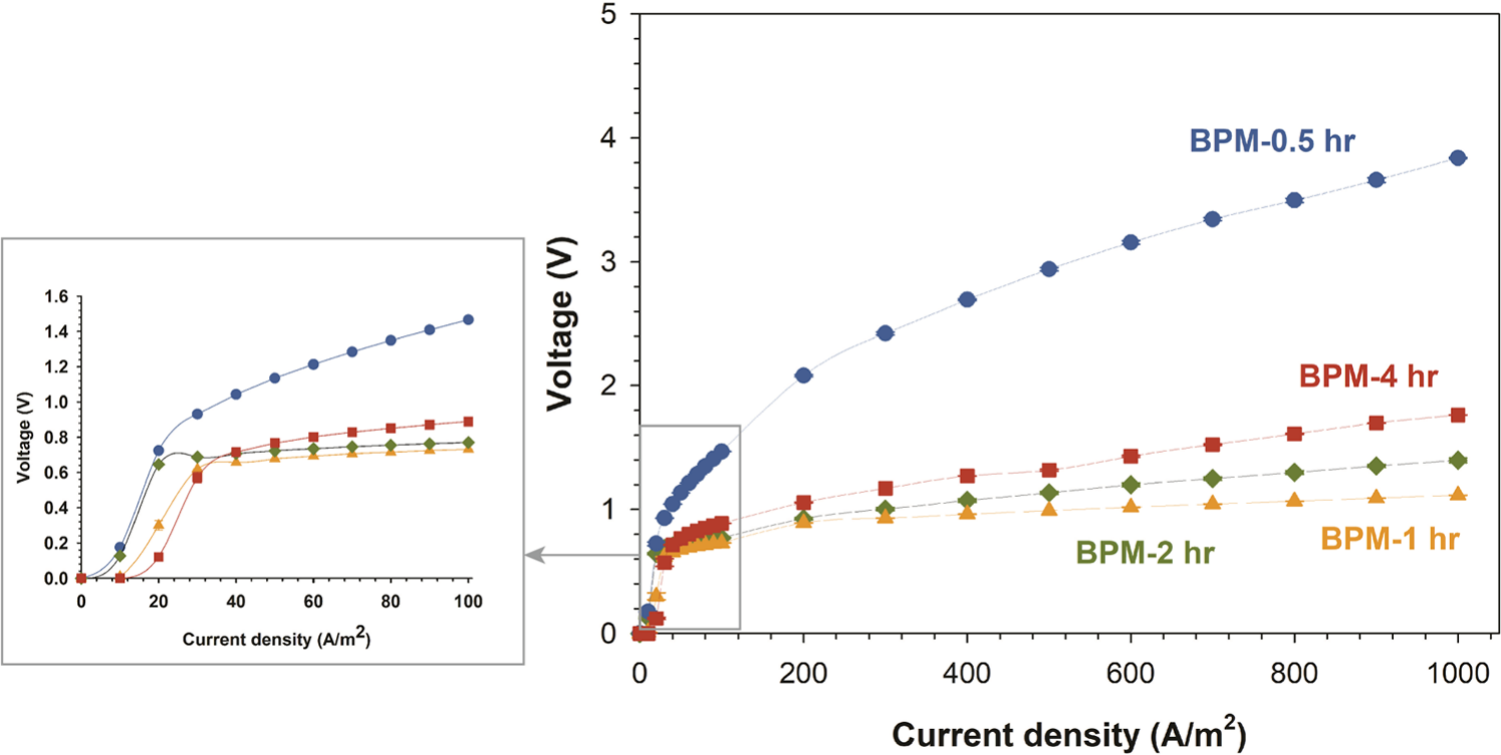
*I*–*V* curves for fabricated electrospun BPMs with MCM-41 catalyst introduced at both sides of the 3D junction, characterized in 1 M NaCl solution over an extended current density range up to 1000 A/m^2^ (zoomed out *I*–*V* curve narrow range up to 100 A/m^2^); error bars are present but are typically smaller than the markers. Errors are for triple *I*–*V* curve measurement for the same fabricated BPM.

Galvanostatic polarization curves (measuring the transmembrane voltage vs current density) were measured for all fabricated BPMs after fabrication and conditioning in a homemade PMMA five-compartment cell with 1 M NaCl as an electrolyte solution. In this study, a single BPM was fabricated for each BPM type, while multiple cycles of electrochemical characterization were conducted for each membrane. During conditioning, fabricated BPM is immersed in 1 M NaCl solutions for roughly 24 h, and then the electrolyte solution is circulated across the BPM after fixing the BPM into the testing cell. Prior to the start of the testing, a total of three IV curves were applied. Water dissociation curves assess BPMs’ ability to dissociate water at various current densities, while voltage response provides information about the energy consumed for acid/base generation. Figure 5 presents the *I*–*V* curves for the four BPMs with different amounts of MCM-41 catalyst introduced on both sides of the 3D junction (BPM-0.5 to BPM-4 h). We observe the plateau of the IV curves associated with the limiting current density, starting at different points between current densities of approximately 20 and 30 A/m^2^. Several factors affect this behavior, such as the composition of the ion exchange materials and the concentration of the salt ions, as discussed in our previous work.^6^ In this work, only the concentration of the catalyst (MCM-41) is varied while fixing all other parameters of materials.

By comparing the *I*–*V* curves of the BPM in the extended current density range as a measure of their electrochemical performance, BPM-0.5 h showed the highest transmembrane voltage of 3.84 V at 1000 A/m^2^, indicating a low water dissociation performance. Notably, the main difference among the BPMs IV curves shown in Figure 5 is the loading of MCM-41 nanoparticles introduced during the fabrication, as all of the other manufacturing parameters were kept constant. Here, BPM-1 h exhibited the best performance of water dissociation with an overpotential of only 280 mV (at 1000 A/m^2^) above the theoretical water dissociation limit of 0.83 V. By comparison, electrospun BPM with the same 3D junction structure but with no added MCM-41 catalyst had a performance of water dissociation with a voltage exceeding 5 V at 1000 A/m^2^ (in 0.5 M NaCl).^7^ However, increasing the catalytic nanoparticle loading (MCM-41) did not necessarily result in an improvement in the performance. Indeed, the overpotential of BPM-4 h with a loading of 0.53 mg/cm^2^ was 3-fold higher than for BPM-1 h with a catalyst loading of 0.13 mg/cm^2^. It is evident from the *I*–*V* curves of the BPMs that the introduction of MCM-41 has a favorable catalytic effect, lowering the energy barrier for the water dissociation reaction in BPM. Therefore, the specific area resistance of BPM-1 h is the lowest (3 Ω·cm^2^) compared to the remaining fabricated BPMs (listed in Table 4), while it is also lower than the benchmarked BPM Fumasep (at 5 Ω·cm^2^). However, the MCM-41 catalyst loading has an optimal range that delivers the best performance. That is observed from the sharp drop of the ohmic resistance when doubling the catalyst loading (from BPM-0.5 h to BPM-1 h); in this case, catalyst loading approaches an optimal range. However, with additional increase of the catalyst loading, the ohmic resistance increases but still remains substantially lower than that of the lowest loading (and blank BPM). The appearance of an optimal water splitting catalyst loading phenomenon has been previously reported^47−49^ and is mostly attributed to the catalyst shadowing effect, where increasing the loading above the optimum negatively impacts the contact active sites of AEL/CEL, dictates the catalyst accessibility, and hinders the transport of reactants (water and ions).

**Table 4:** Fabricated BPMs Resistance in 1 M NaCl

	specific area resistance in 1 M NaCl
BPM	Ω·cm^2^
BPM-0.5 h	23
BPM-1 h	3
BPM-2 h	6
BPM-4 h	9
BPM-1 h-CES	28
BPM-1 h-AES	7
Fumasep BPM	5

**Figure 6:**
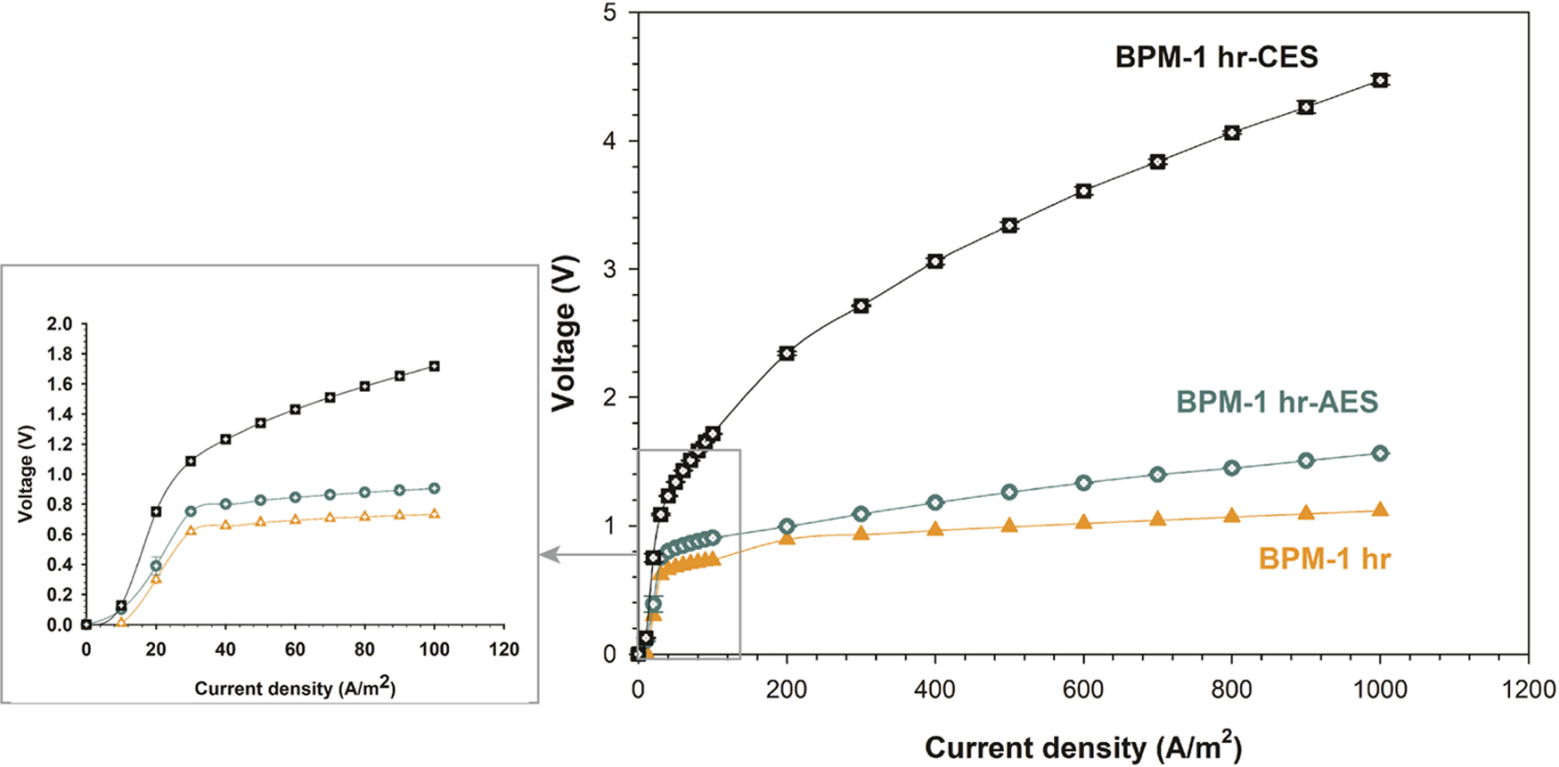
Water dissociation *I*–*V* curves across fabricated electrospun BPMs with MCM-41 catalyst introduced only at one side of the 3D junction compared with BPM-1 h, characterized in 1 M NaCl solution over extended current density range up to 1000 A/m^2^ (zoomed out *I*–*V* curve for narrow current density range up to 100 A/m^2^); error bars are present but are typically smaller than the markers. Errors are for triple *I*–*V* curve measurement for the same fabricated BPM.

In order to evaluate the effect of the catalyst location in the structure of the BPM, two more BPMs were fabricated, namely, BPM-1 h-CES and BPM-1 h-AES. In this case, MCM-41 nanoparticles were electrosprayed to one predefined side of the 3D junction: to the cation exchange side for BPM-1 h-CES and to the anion exchange side in the case of BPM-1 h-AES, with a fixed catalyst loading (0.07 mg/cm^2^) in both situations. Figure 6 shows the electrochemical performance of BPMs up to a current density of 1000 A/m^2^. The transmembrane voltages were recorded being 4.47 and 1.56 V at 1000 A/m^2^ for BPM-1 h-CES and BPM-1 h-AES, respectively. This represents a drastic electrochemical performance difference for BPMs with the same catalyst loading. Such an observation matches with the findings of Oener et al.,^25^ where they have extensively investigated several metal-based water dissociation catalysts. They studied the effect of the alkaline environment of the AEL and the acidic environment of the CEL on the performance of the water dissociation catalyst. Our findings clearly indicate that MCM-41 is a much more efficient water dissociation catalyst in an alkaline environment (in the AEL). Still, the performance of the membrane with catalysts on both sides of the junction is better than the performance of the membrane with a catalyst only in the AEL, demonstrating that there is still a role for MCM-41 in the CEL. Potentially, another catalyst could be introduced in the CEL to further improve the membrane, but that goes beyond this current investigation.

**Figure 7:**
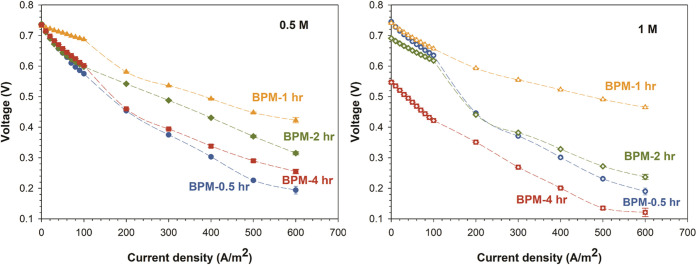
BPM voltage drop across all of the investigated BPMs during water association at different HCl and NaOH concentrations of 0.5 and 1 M in a current density range of 0–600 A/m^2^. Error bars are present but are typically smaller than the markers.

### 3.3 Open-Circuit Voltage (OCV)

Open-circuit voltages (OCVs) of the fabricated BPMs are shown in Table 5, as measured with continuously flowing HCl (0.5 and 1 M solution) at the cation exchange side of the BPM and NaOH (0.5 and 1 M solution) at the anion exchange side of the BPM. OCV measurements provide an insight into the behavior of BPM in maintaining a potential of the bipolar membrane with the acid (HCl)/base (NaOH) solutions in relation to BPM performance in flow batteries and fuel cell systems. The OCV is measured using electrodes (Haber–Luggin capillary) adjacent to the BPM. As a result, measuring the voltage across BPM would coincide with measuring the voltage change across BPM. Initially, OCV is the potential that exists throughout the BPM as a result of the flowing of acid and basic solutions. Based on the concentration of the solutions, this potential is determined using the Nernst equation.^9^ As a result of this, any voltage drop for the recorded values in the table will be mostly caused by unwanted leakage of protons (H^+^) and hydroxide ions (OH^–^).

**Table 5:** Open-Circuit Voltage (OCV) Values across All Fabricated BPMs at Two Different HCl-NaOH Concentrations of 0.5 M HCl and NaOH and 1 M HCl and NaOH

	OCV in 0.5 M HCl/NaOH	original OCV value at 0.5 M	deviation from theoretical (ideal) value	OCV in 1 M HCl/NaOH	original OCV value at 1 M	deviation from theoretical (ideal) value
BPM	V	V	V	V	V	V
BPM-0.5 h	0.74	0.789	0.049	0.75	0.824	0.074
BPM-1 h	0.73	0.789	0.059	0.75	0.824	0.075
BPM-2 h	0.73	0.789	0.055	0.72	0.824	0.1
BPM-4 h	0.74	0.789	0.052	0.72	0.824	0.101
BPM-1 h-AES	0.73	0.789	0.058	0.72	0.824	0.109
BPM-1 h-CES	0.62	0.789	0.168	0.51	0.824	0.310

We observe that the recorded OCVs were between 0.72 and 0.75 V for all fabricated BPMs in this work except for BPM-1 h-CES, where the OCVs were 0.62 and 0.51 V and were measured for solution concentrations of 0.5 and 1 M, respectively. Average values of OCVs for the fabricated BPMs in this work are higher (closer to theoretical values of 0.79 V for 0.5 M HCl and NaOH and 0.83 V for 1 M HCl and NaOH @ 25 °C) than the values reported in our previous work with similarly structured membranes.^7,50^ This is mostly an indication of the positive effects of having MCM-41 as the water dissociation catalyst, where it could impact both the ion selectivity of the ion exchange layers and the recombination rate of H^+^/OH^–^ at the BPM junction.^51−53^ We also again see that MCM-41 works less well under acidic conditions; hence, the lower voltage for BPM-1 h-CES. Furthermore, the OCV data show an improvement due to utilization of MCM-41 as the water dissociation/formation catalyst, except for BPM-1 h-CES, where addition of MCM-41 to the cation exchange side did not improve the OCV.

### 3.4 Water Association Electrochemical Characterization

The BPMs were then evaluated in forward bias mode (water association mode). The voltage drops across the BPMs, referring to the lower magnitude of the negative overpotential, with MCM-41 introduced on both sides, are presented in Figure 7. However, lower voltage drop in this case relates to lower energy dissipation (higher H^+^–OH^–^ recombination and lower crossover). Operating BPM in forward bias mode is important for their application in fuel cells,^54^ redox-flow batteries,^6^ and CO_2_ reduction.^55^ BPMs exhibited a lower voltage drop in comparison with pristine BPMs (not including MCM-41) as reported in our previous work.^6,50^ Moreover, we again see a clear optimum for the membrane with 1 h of catalyst deposition, in line with the earlier observed optimum. Clearly, adding MCM-41 as a catalyst using our unique hybrid electrospinning/electrospraying approach leads to enhanced performance in water splitting and in acid and base recombination.

**Figure 8:**
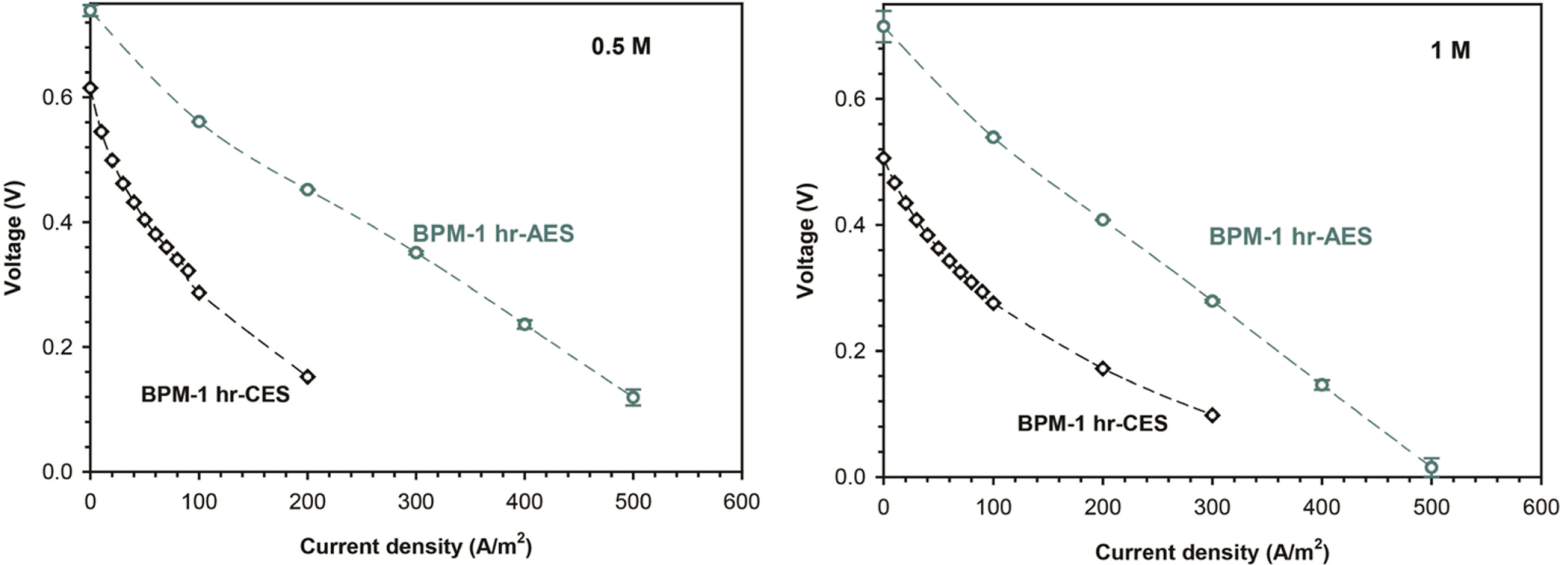
BPM voltage drop across the investigated BPMs (with MCM-41 incorporated in either side of the junction) during water association at different HCl and NaOH concentrations of 0.5 and 1 M NaCl at the current density range of 0–500 A/m^2^. Error bars are present but are typically smaller than the markers.

Mitchell et al.^51^ have reported that oxide nanoparticles promote recombination rate of H^+^/OH^–^, and they hypothesized that the enhanced recombination rate is due to the polyprotic oxide surfaces. Such surfaces facilitate H^+^ and OH^–^ recombination by enabling intermediate proton transfer to/from a large surface area, eliminating the necessity for direct H^+^/OH^–^ recombination.^51,56^ We find that the results of our research support their conclusions. MCM-41 possesses catalytic activity during water association in a forward bias mode, comparable to the effect during water dissociation in a reverse bias mode.

Additionally, the water association performance of BPMs with MCM-41 nanoparticles incorporated only to one side of the junction (BPM-1 h-AES and BPM-1 h -CES) was assessed, as shown in Figure 8. The aspect of placing the catalyst nanoparticles on either side of the junction (AES/CES) influences the water association findings (voltage drop). As with the previous observations, MCM-41 incorporation into AEL resulted in a superior performance when compared to the alternative of integrating the same catalyst loading into the CEL. Accordingly, as with the previous results (Figure 6), the performance of both BPMs in the forward bias mode corresponds to their performance in the reverse bias mode.

### 3.5 Current Efficiency and Energy Consumption

**Figure 9:**
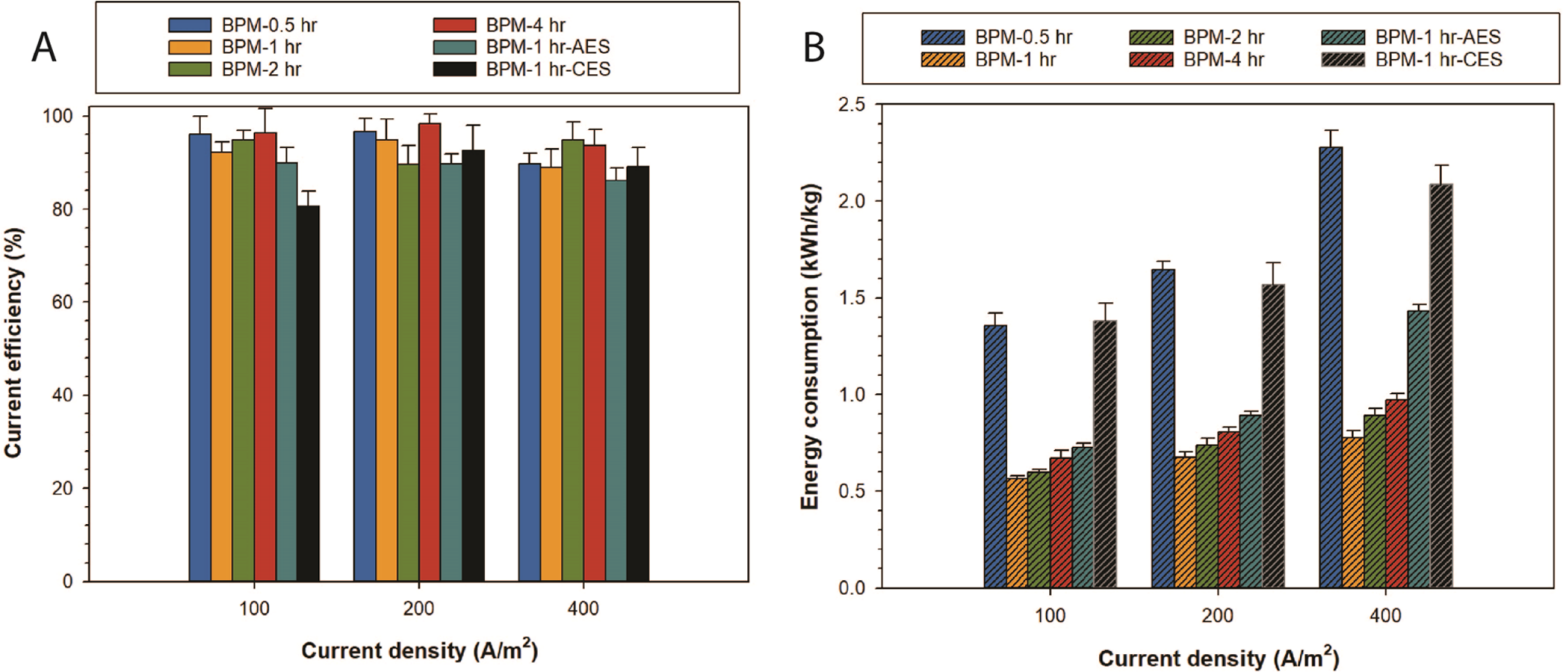
(A) Current efficiency (%) of all BPMs at three current densities of 100, 200, and 400 A/m^2^ tested in 0.1 M NaCl solution. (B) Specific energy consumption (in kWh/kg) for producing an equivalent of 1 kg of NaOH/HCl using fabricated BPMs at different current densities of 100, 200, and 400 A/m^2^.

Current efficiency is assessed by the volume of HCl/NaOH produced at a specific current density; it has been evaluated for the manufactured BPMs in 0.1 M NaCl solution at varied current densities of 100, 200, and 400 A/m^2^. Figure 9 shows the current efficiencies evaluated for the fabricated BPMs, and in all cases, the current efficiencies are higher than 90%, in alignment with the promising results of BPMs performance found during water dissociation and formation.

Notwithstanding, BPM-1 h-CES had, on average, a lower current efficiency, which is mostly attributed to the lower activity of MCM-41 in the acidic environment of the cation exchange layer. Overall, the role of MCM-41 integration in improving product purity is minimal (i.e.,: HCL and NaOH), as observed in Figure 9A. Although operating current density, BPM monolayer permselectivity, and bulk salt concentration are the key parameters influencing current efficiency,^6,57^ a superior accelerating water dissociation catalyst would assist to start achieving higher current efficiencies at lower current densities of operation. This would widen the operating current range at which the BPM would efficiently operate with maximum capacity. In comparison with the function of BPMs with no catalysts, higher current densities would be needed to achieve high current efficiencies.^6^

In Figure 9B, energy consumption data for producing an equivalent of 1 kg_eq_ of NaOH are presented for fabricated BPMs, as estimated from eq 2. Noteworthy, we observe that the energy consumption increases slightly when ramping up the operating current density. Moreover, despite recording a better water dissociation performance, BPM-1 h shows less current efficiency compared with other BPMs (even with lower performance). From the observations of the performance of all fabricated BPMs, the correlation between water dissociation performance and current efficiency is dependent on many factors including but not limited to current density and selectivities of monopolar layers.

BPM-1 h utilizes the lowest specific energy for generating acid/base, with an energy consumption of 0.56–0.78 kWh/kg_eq_ (0.022–0.031 kWh/mol) measured for the case of BPM-1 h as the lowest reported in this research field. For comparison, we found Fumasep BPM to have a higher energy consumption of 1.89 kWh/kg_eq_ at 100 A/m^2^ (0.0756 kWh/mol) when tested under the same conditions. It is important to note that there are differences in limiting current density between Fumasep BPM and the fabricated ones in this work. As a result of that, the energy consumption spread would likely decrease at higher current densities. The big difference of energy consumption with the commercial BPM mainly arises from the higher limiting current density of operation for Fumasep BPM in comparison with the developed BPM in this research. Reported energy consumptions of NaOH and HCl production with developed and commercial membranes vary significantly based on various factors, including applications, process conditions, and initial salt (NaCl) concentrations. Table 6 summarizes some of the available literature data on BPM energy consumption for NaOH and HCl production.

**Table 6:** Some Literature Data on Energy Consumption of Acid–Base Production with BPM

specific energy consumption (kWh/kg)	current density (A/m^2^)	application	reference
0.56–0.78	100–400	NaOH and HCl production	this work
2.3–18	300–1100	EDBM with brine for NaOH and HCl production	(58)
2.73	600	BPM developed with graphene oxide	(59)
1.54–2.33	∼500–600	BMED for mixed saltwater treatment	(60)
2.16	∼60	developed BPM with incorporated nano-MoS_2_ interfacial layer	(61)

## 4 Conclusions

A novel approach of hybrid electrospinning/electrospraying for BPM fabrication has been proposed in this work. By coupling the electrospinning of ion exchange polymers and electrospraying of dispersed catalyst nanoparticles, we enabled production of BPMs with a high stability, an enlarged junction specific surface area, and the flexibility of introducing a variety of catalyst materials. Additionally, the incorporation of a new porous catalyst material of MCM-41 (silica nanoparticles) has been explored as the primary water dissociation catalyst in the BPM.

Several BPMs with varied catalyst (MCM-41) loadings were developed in this work by depositing MCM-41 silica nanoparticles in layers directly adjacent to the BPM 3D junction and also by depositing MCM-41 silica nanoparticles solely on the cation exchange side or the anion exchange side of the junction.

The water dissociation current–voltage curves of the newly developed bipolar membranes demonstrated a significant improvement in the BPM electrochemical performance through lowered BPM transmembrane voltage. The BPM with the optimal catalyst (MCM-41) loading (0.13 mg/cm2) recorded an overpotential of 280 mV at 1000 A/m^2^, exceeding the performance of the best reported benchmarked commercial BPM of Fumasep. Fabricated BPM-2 h water dissociation performance is compared to commercial BPM Fumasep (see the Supporting Information, Figure S1). Our fabricated BPMs have lower recorded voltage and better water dissociation performance across the BPM. Furthermore, the data of open-circuit voltage and water formation characterization clearly highlight the benefit of the catalyst when operating the BPM at the forward bias mode, whereas the voltage drop still sustains above 0.2 V at 500 A/cm^2^, an important feature for applications of fuel cells and flow batteries.

Advancement in developing BPM has been demonstrated in this work by introducing a novel BPM architecture through hybrid electrospinning–electrospraying and introducing a new catalyst MCM-41. We foresee that the choice of catalyst in BPM specifically designated for the roles of water dissociation and water formation would be the future trend in high-performance BPM development.

## Supporting Information Available

Current–voltage curve is displayed comparing a fabricated BPM from this work, namely, BPM-2 h and Fumasep BPM as a benchmark commercial BPM; characterization is carried out at a current density of 0–500 A/m^2^ and in a five-compartment setup and electrolyte solution of 1 M NaCl at both sides of BPM (PDF)
