# Supplementary material for: Combined Electrospinning–Electrospraying for High-Performance Bipolar Membranes with Incorporated MCM-41 as Water Dissociation Catalysts
Source: ACS Appl Mater Interfaces. 2023 Sep 20;15(39):45745–55. doi: 10.1021/acsami.3c06826 (PMC10561145; doi:10.1021/acsami.3c06826)
Supplement: Supplementary file 1 — am3c06826_si_001.pdf [file am3c06826_si_001.pdf]

Supporting information

Combined electrospinning-electrospraying for high performance bipolar membranes with incorporated MCM-41 as water dissociation catalysts

Emad Al-Dhubhani [a,b], Michele Tedesco [a], Wiebe M. de Vos [b*],Michel Saakes [a]

[a] Wetsus, European Centre of Excellence for Sustainable Water Technology

Oostergoweg 9, 8911 MA Leeuwarden, The Netherlands

[b] Membrane Science and Technology, University of Twente, P.O. Box 217, 7500 AE Enschede, The Netherlands

*Corresponding author (W.M. de Vos): w.m.devos@utwente.nl

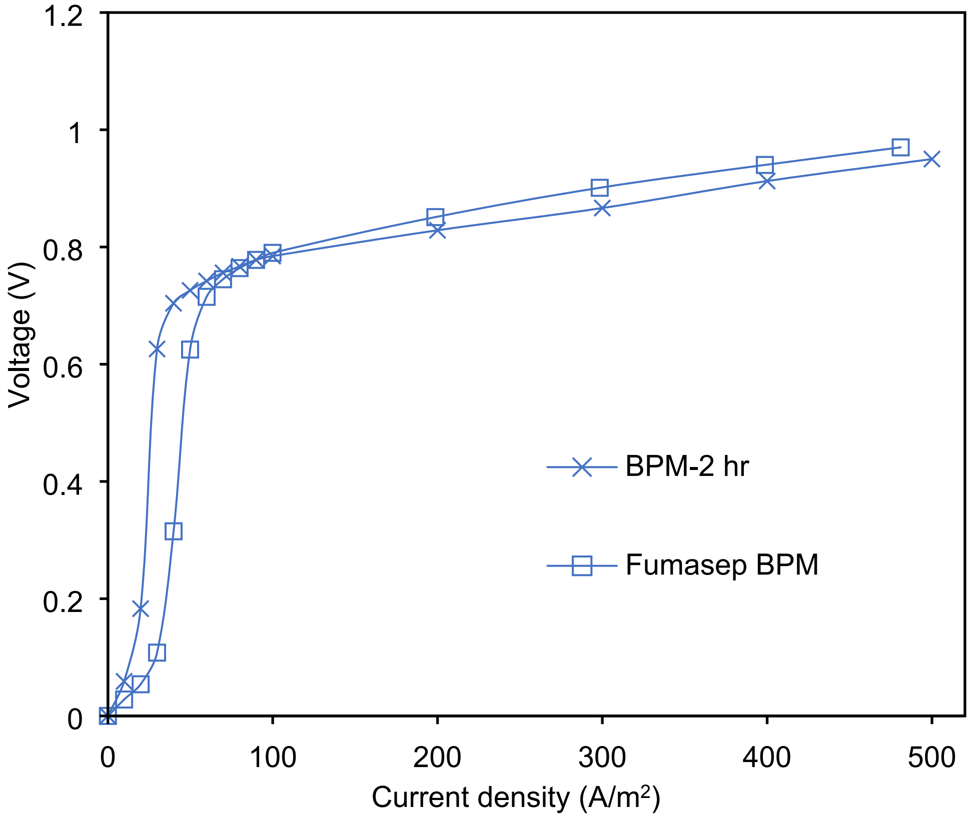

Figure S1: I-V curve performance comparison between developed BPM-2 hr and commercial BPM Fumasep
